# Prognosis and safety of radium‐223 with concurrent abiraterone acetate or enzalutamide use for metastatic castration‐resistant prostate cancer: Real‐world data of Japanese patients

**DOI:** 10.1002/bco2.42

**Published:** 2020-09-05

**Authors:** Yasuhide Miyoshi, Masato Yasui, Sohgo Ttsutsumi, Takashi Kawahara, Ko‐ichi Uemura, Naruhiko Hayashi, Masahiro Nozawa, Kazuhiro Yoshimura, Hiroji Uemura, Hirotsugu Uemura

**Affiliations:** ^1^ Department of Urology and Renal Transplantation Yokohama City University Medical Center Yokohama Japan; ^2^ Department of Urology Graduate School of Medicine Yokohama City University Yokohama Japan; ^3^ Department of Urology Faculty Medicine Kindai University Osaka Japan

**Keywords:** abiraterone acetate, castration‐resistant prostate cancer, enzalutamide, prostate cancer, radium‐223

## Abstract

**Objectives:**

To evaluate the real‐world data on the efficacy and safety of a combination therapy with radium‐223 (Ra‐223) and second‐generation androgen‐receptor targeting agents (ARTAs), including abiraterone acetate (ABI) and enzalutamide (ENZ), among Japanese patients with bone metastatic castration‐resistant prostate cancer (CRPC).

**Patients and methods:**

We retrospectively reviewed 79 patients with bone metastatic CRPC who were treated with Ra‐223. The number of patients with concurrent ARTA use was 24:17 receiving ABI and 7 receiving ENZ. We evaluated the overall survival (OS) according to ARTA use and compared the survival of patients treated with Ra‐223 with or without ARTA using multivariate analysis.

**Results:**

The median survival in the entire cohort was 23.5 months. The patients receiving Ra‐223 combined with ARTA showed a tendency of better OS than patients treated with Ra‐223 alone, although no significant difference was observed (median OS, 26.5 vs 23.5 months; *P* = .115). A multivariate analysis showed that the extent of disease on bone scan (EOD) scores and pain at baseline were significant predictors of OS. The concurrent use of bone‐modifying agents (BMAs) was not significant for favorable OS (*P* = .050). However, the concurrent use of second‐generation ARTA was not a significant factor for OS. Regarding safety, a bone fracture occurred in only one (4.2%) of 24 patients treated with combined Ra‐223 and ARTA therapy.

**Conclusion:**

Our real‐world data analysis suggested that Ra‐223 combined with a second‐generation ARTA is well tolerated in Japanese patients. The EOD score and pain at baseline are significant prognostic factors for OS, but the concurrent use of second‐generation ARTA has no influence on OS among men treated with Ra‐223. The concurrent use of BMA yields a marginally favorable OS.

## 1 INTRODUCTION

The treatment of metastatic castration‐resistant prostate cancer (mCRPC) has improved dramatically in the past few decades. After their approval, the new agents docetaxel,^1^ cabazitaxel,^2^ sipuleucel‐T,^3^ radium‐223 (Ra‐223),^4^ olaparib,^5^ and second‐generation androgen‐receptor targeting agents (ARTAs), including abiraterone acetate (ABI)^6^, ^7^ and enzalutamide (ENZ),^8^, ^9^ are now considered as the mainstay of treatment for mCRPC.

As each of these drugs showed efficacy in monotherapy, their combination therapies were expected to show more survival benefit. However, in a randomized, double‐blind, placebo‐controlled, phase III ERA 223 trial,^10^ the addition of Ra‐223 to ABI showed no improvement over survival and rather resulted in an increased frequency of bone fractures compared with ABI alone. Following these results of the ERA 223 trial, the European medicines agency (EMA) issued a formal warning against the use of Ra‐223 in combination with ABI for mCRPC in March 2018.^11^ Subsequently, the United States Food and Drug Administration^12^ and the Pharmaceuticals and Medical Devices Agency, Japan,^13^ instructed the avoidance of this combination.

Since then, this combination therapy is not being used. However, certain studies have shown the effectiveness and safety of this combination therapy.^14^, ^15^, ^16^ Thus, we investigated the real‐world data on the efficacy and safety of a combination therapy of Ra‐223 and a second‐generation ARTA for mCRPC among Japanese patients who were treated before the EMA issued a warning.

## 2 PATIENTS AND METHODS

### 2.1 Patients

We retrospectively reviewed the data of 79 patients with mCRPC metastasized to bone and who were treated with Ra‐223 between 2012 and 2017 at the Yokohama City University Medical Center and Kindai University Hospital. Some patients participated in the Japanese trial of Ra‐223. All patients had histologically confirmed prostate adenocarcinoma at the initial diagnosis. The patients with distant metastases except bone and/or with regional lymph node metastases greater than 4 cm were excluded from this study. Ra‐223 was administered at a dose of 55 kBq (1.49 microcurie)/kg intravenously every 4 weeks up to six cycles. In addition, the luteinizing hormone‐releasing hormone (LHRH) agonist or antagonist was administrated to all patients. After Ra‐223 failed, all patients continued to receive LHRH agonist or antagonist and were subsequently treated according to each physician's treatment strategy. For terminally ill patients, palliative therapy and pain control with morphine and palliative external‐beam radiation were used, as appropriate.

Ra‐223 combined with ARTA was used in 24 patients from the start of therapy; the ARTA included ENZ 160 mg/day (seven patients) and ABI 1,000 mg with prednisone 10 mg/day (17 patients) (combination group). The remaining 55 patients were treated with Ra‐223 without ARTA (single group).

### 2.2 Clinical assessments

The primary endpoint of this study was survival in the combination group compared with that in the single group. According to the ERA 223 results, we investigated the frequency of bone fracture only in the combination group because we were concerned about the increased frequency of bone fracture in the patients who received Ra‐223 concurrently with ARTA. Therefore, the frequency of bone fracture in the single group could not be evaluated. The secondary endpoint was the frequency of bone fractures in the combination group.

Patient's disease state was clinically staged according to the 1997 TNM classification by computed tomography and bone scan using ^99^mTc‐methylene diphosphonate. The extent of bone metastases evaluated by the bone scan was classified according to the extent of disease on bone scan (EOD) score, as suggested by Soloway et al.^17^

Eleven variables at baseline were assessed from the electronical medical records; these included patient age, time to CRPC from initial androgen‐deprivation therapy, previous use of docetaxel, prostate‐specific antigen (PSA) level, hemoglobin level, lactate dehydrogenase level, alkaline phosphatase (ALP) level, Gleason scores (GS), EOD scores, concurrent use of bone‐modifying agents (BMAs), and pain at baseline. Denosumab 120 mg was administrated every 4 weeks and zoledronic acid 4 mg was administrated every 4 weeks to patients treated with BMAs. Pain at baseline wad defined as “patient‐reported complaint” with or without painkiller use. In Japan, Ra‐223 has been approved for not only mCRPC patients with pain, but also those without pain. GS was determined according to The 2005 International Society of Urologic Pathology Gleason grading.^18^ Serum PSA levels were measured using the Elecsys PSA Assay (Roche Diagnostics, Basel, Switzerland). The occurrence of bone fracture after Ra‐223 administration in the combination group was evaluated by CT and X‐ray radiography. The patients were followed up every 4 weeks in the outpatient setting.

### 2.3 Statistical analysis

Differences in patient characteristics between the combination group and the single group were analyzed by Mann‐Whitney *U* test and chi‐square test. Overall survival (OS) was defined as the time between initiation of Ra‐223 treatment to the final date of consultation or death. A Kaplan‐Meier product‐limit estimator was used to assess OS distribution. Log‐rank tests were used to analyze differences in OS between the two groups. Univariate and multivariate Cox proportional hazard models were used to analyze prognostic factors of OS. Moreover, relative risks and 95% confidence intervals (95% CIs) were determined. All tests were two‐sided; *α* = .05 was considered significant. All analyses were conducted using the IBM SPSS Statistics software for Windows, version 22 (IBM Corp., Armonk, NY, USA) and EZR (Saitama Medical Center, Jichi Medical University, Saitama, Japan).^19^

Experimental procedures were conducted in accordance with the ethical standards of the Helsinki Declaration. This study was approved by the institutional review board of Yokohama City University Medical Center (B181000040). Informed consent to participate in the study was obtained from all subjects in an opt out manner.

## 3 RESULTS

The median observation period was 12.5 months and the number of the patients who were lost to follow‐up was five.

The characteristics of patients in the combination group and single group are shown in Table 1. The number of patients who previously used docetaxel was 31 (39.2%), and the remaining 48 (60.8%) patients received Ra‐223 before docetaxel. BMAs were administered to 31 patients (39.2%) in the entire cohort. Of those 31 patients, 16 and 15 patients received denosumab and zoledronic acid, respectively.

**TABLE 1:** Characteristics of patients

	All cohort (n = 79)	Combination group (n = 24)	Single group (n = 55)	*P*‐value
Median age, years (range)	75 (49‐88)	75 (61‐88)	74 (49‐85)	0.277
Median time to CRPC, months (range)	13 (0‐155)	19.0 (0‐109)	12 (2‐155)	0.503
Previous use of docetaxel, n (%)	31 (39.2)	7/24 (29.2)	24/55 (43.6)	0.317
Median PSA, ng/mL (range)	38.9 (0.0‐1795.0)	19.7 (0.0‐629.0)	47.1 (0.0‐1795.0)	0.035*
Median Hemoglobin, g/dL (range)	12.6 (7.5‐15.5)	12.8 (7.5‐15.2)	12.2 (8.0‐15.5)	0.073
Median LDH, IU/L (range)	217 (133‐528)	201 (140‐512)	224 (133‐528)	0.321
Median ALP, IU/L (range)	324 (113‐3540)	243 (142‐1939)	404 (113‐3540)	0.026*
Gleason scores 6‐7, n (%)	15 (19.0)	6/24 (25.0)	9/55 (16.4)	0.370
Gleason scores 8‐10, n (%)	64 (81.0)	18/24 (75.0)	46/55 (83.6)	
EOD 1‐2, n (%)	50 (63.3)	18/24 (75.0)	32/55(58.2)	0.207
EOD 3‐4, n (%)	29 (36.7)	6/24 (25.0)	23/55(41.8)	
Concurrent use of BMA	31 (39.2)	10/24 (41.7)	21/55 (38.2)	0.806
Pain (%)	43 (54.4)	10/24 (41.7)	33/55 (60.0)	0.149

Abbreviations: ALP, alkaline phosphatase; ARTA, androgen receptor‐target agents; BMA, bone‐modified agent; CRPC, castration‐resistant prostate cancer; EOD, extent of disease on bone scan; LDH, lactate dehydrogenase; PSA, prostate‐specific antigen.

* Indicates statistical significance (*P* < .05).

The PSA levels (median 19.7 ng/mL; range 0.0‐629.0 ng/mL in the combination group, median 47.1 ng/mL; range 0.0‐1795.0 ng/mL in the single group, *P* = .035) and ALP levels (median 243 IU/l; range 142‐1939 IU/l in the combination group, median 404 IU/l; range 113‐3540 IU/l in the single group, *P* = .026) were significantly higher in the single group. Overall, 60 (75.9%) patients received ≥5 cycles of Ra‐223:22/24 (91.7%) in combination group and single group: 38/55 (69.0%) in single group. The patients without the concurrent use of ARTA were less likely to receive ≥5 cycle of Ra‐223.

**FIGURE 1:**
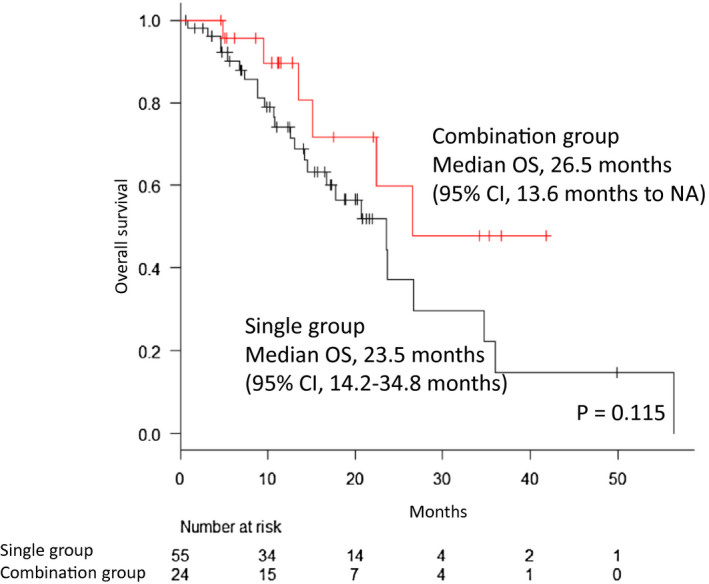
Kaplan‐Meier curve for overall survival (OS) of patients treated with radium‐223 along with a second‐generation androgen‐receptor targeting agent (ARTA). The red line shows the survival curve for the patients treated with Ra‐223 and ARTA (combination group), and the black line shows the survival curve for patients treated by Ra‐223 alone (single group). The median OS for patients in the combination group and single group was 26.5 and 23.5 months, respectively. CI, confidence interval; NA, not available

The median OS in all cohorts was 23.5 months (95% CI, 16.7‐34.8). Comparison of OS between the combination group and single group is shown in Figure 1. The median OS was 26.5 (95% CI, 13.6‐not available [NA]) and 23.5 (95% CI, 14.2‐34.8) months in the combination group and single group, respectively. There tendency of better OS was observed in the combination group, albeit without statistical significance between the two groups (*P* = .115). The Kaplan‐Meier curve for OS according to the concurrent use of ABI and ENZ is shown in Figure S1. The median OS was not reached (95% CI, 4.8 months to NA) and 26.5 (95% CI, 13.6‐NA) months by patients concurrently treated with ENZ and ABI, respectively.

**FIGURE 2:**
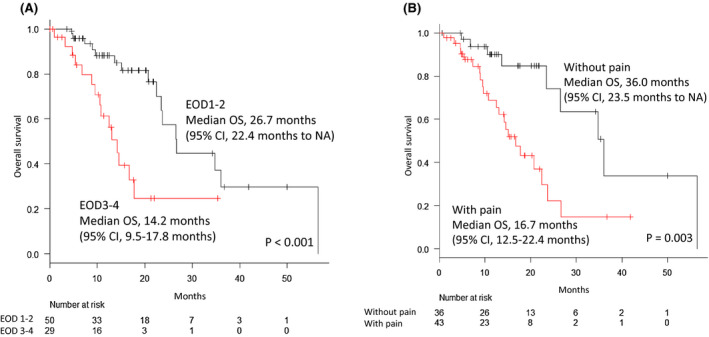
A, Kaplan‐Meier curve for the overall survival (OS) of patients treated with radium‐223 according to the extent of disease on bone scan (EOD) scores. The red line shows the survival curve for the patients with EOD score 3‐4, and the black line shows the survival curve for the patients with EOD score 1‐2. The median OS for patients with EOD scores 1‐2 and 3‐4 was 26.7 and 14.2 months, respectively. B, Kaplan‐Meier curve for the overall survival (OS) of patients treated with radium‐223 according to the pain at baseline. The red line shows the survival curve for the patients with pain, and the black line shows the survival curve for the patients without pain. The median OS for patients without and with pain was 36.0 and 16.7 months, respectively. CI, confidence interval; EOD, extent of disease on bone scan; NA, not available

The Kaplan‐Meier curve for OS according to EOD score and pain at baseline is shown in Figure 2A,B. The median OS was 26.7 (95% CI, 22.4‐NA) and 14.2 (95% CI, 9.5‐17.8) months for patients with EOD score 1‐2 and 3‐4, respectively (Figure 2A). The median OS was 36.0 (95% CI, 23.5‐NA) and 16.7 (95% CI, 12.5‐22.4) months for patients without pain and with pain, respectively (Figure 2B). Patients with both EOD score 3‐4 and pain at baseline had shorter OS than those with EOD score 1‐2 and without pain (*P* < .001 and *P* = .003, respectively).

The results of univariate and multivariate analyses to identify the risk factors for OS are shown in Table 2. In the univariate analysis, PSA level (> median vs ≤ median, hazard ratio [HR]: 3.65, 95% CI: 1.65‐8.07, *P* = .001), hemoglobin level (<median vs ≥ median, HR: 2.44, 95% CI: 1.16‐5.12, *P* = .019), ALP level (> median vs ≤ median, HR: 3.01, 95% CI: 1.37‐6.63, *P* = .006), EOD score (3‐4 vs 1‐2, HR: 3.49, 95% CI: 1.64‐7.45, *P* = .001), and pain (yes vs no, HR: 3.28, 95% CI: 1.45‐7.44, *P* = .004) were significantly related to the OS. The multivariate analysis showed that both EOD scores (3‐4 vs 1‐2, HR 4.98, 95% CI: 1.46‐16.96, *P* = .010) and pain (yes vs no, HR 4.40, 95% CI: 1.22‐15.90, *P* = .024) were the significant predictors for OS. In contrast, patients not receiving concurrent BMAs (nonuse of BMA vs use, HR 3.50, 95% CI: 1.00‐12.26, *P* = .050) had a tendency of worse OS compared with patients receiving BMA but without statistical significance. Furthermore, the concurrent use of a second‐generation ARTA (yes vs no, HR: 0.66, 95% CI: 0.22‐2.02, *P* = .470) had no influence on OS.

**TABLE 2:** Univariate and multivariate analyses for predicting overall survival of men treated with radium‐223

	Univariate				Multivariate			
	*P*‐value	HR	95% CI		*P*‐value	HR	95% CI	
			Lower	Upper			Lower	Upper
Age > median vs ≤ median	0.547	0.80	0.39	1.65	0.104	0.44	0.16	1.19
Time to CRPC < median vs ≥ median	0.998	1.00	0.48	2.08	0.157	0.44	0.14	1.37
Previous docetaxel use yes vs no	0.652	0.83	0.37	1.87	0.209	0.46	0.14	1.54
PSA > median vs ≤ median	0.001*	3.65	1.65	8.07	0.218	1.99	0.67	5.92
Hemoglobin < median vs ≥ median	0.019*	2.44	1.16	5.12	0.088	2.50	0.87	7.17
LDH > median vs ≤ median	0.211	1.60	0.77	3.35	0.853	1.10	0.39	3.11
ALP > median vs ≤ median	0.006*	3.01	1.37	6.63	0.283	1.71	0.64	4.55
Gleason score 8‐10 vs 6‐7	0.465	0.71	0.28	1.79	0.363	0.56	0.16	1.95
EOD 3‐4 vs 1‐2	0.001*	3.49	1.64	7.45	0.010*	4.98	1.46	16.96
Concurrent nonuse of BMA vs use	0.251	0.65	0.31	1.36	0.050	3.50	1.00	12.26
Pain, yes vs No	0.004*	3.28	1.45	7.44	0.024*	4.40	1.22	15.90
Concurrent use of second generation ARTA vs nonuse	0.121	0.49	0.20	1.21	0.470	0.66	0.22	2.02

Abbreviations: ALP, alkaline phosphatase; ARTA, androgen receptor‐target agent; BMA, bone‐modified agent; CRPC, castration‐resistant prostate cancer; EOD, extent of disease on bone scan; LDH, lactate dehydrogenase; PSA, prostate‐specific antigen.

* Indicates statistical significance (*P* < .05).

As for the secondary endpoint, one of 24 patients (4.2%) treated with combination therapy of Ra‐223 and ABI reported a bone fracture (grade 3). This patient had a metatarsal fracture and thoracic spine compression fracture regardless of the use of BMA. These lesions were not correlated with bone metastases from the prostate cancer.

There was not any specific adverse event in the combination group.

## 4 DISCUSSION

The ALSYMPCA trial demonstrated significant improvements with Ra‐223 regarding the OS, the time to symptomatic skeletal events, and the quality of life compared with placebo in patients with bone metastatic CRPC.^4^, ^20^ The results of this trial led to the incorporation of Ra‐223 as an important treatment strategy in bone metastatic CRPC. Moreover, Saad et al conducted a phase IIIb trial after the ALSYMPCA trial and suggested that concurrent ARTA use with Ra‐223 improved OS.^14^ They reported that 189 (27%) patients in their trial, who received Ra‐223 concurrently with an ARTA, showed significantly favorable OS compared with the 507 (73%) patients who received Ra‐223 alone (median OS; not reached vs 13 months).

Theoretically, Ra‐223 only treats bone metastasis. ARTA could treat metastatic sites other than bone, thus, a combination therapy would be ideal. Indeed, there have been reports of many patients who received Ra‐223 combined with ARTA in a real‐world setting.^15^, ^21^ Sartor et al reported that the OS of patients who received Ra‐223 with ARTA, including ABI or ENZ, (40/184, 21.7%) was favorable compared with that of patients who received only Ra‐223 (144/184, 78.3%) in a phase II, U.S. expanded access program.^15^ Shore et al also conducted a database retrospective analysis of the efficacy and safety of Ra‐223 combined with ARTA in 625 patients with Mcrpc.^21^ Of the 625 patients, 83 (13.3%) received Ra‐223 with ARTA (39 received ABI and 44 received ENZ) and the remaining 542 (86.7%) patients received Ra‐223 alone. The median OS of patients receiving concurrent ABI and concurrent ENZ was 22.1 and 19.1 months, respectively.

However, the results of the ERA 223 trial were against the expectations. The addition of Ra‐223 to ABI failed to show any improvement in the symptomatic skeletal event‐free survival (SSE). The Median SSE of patients in the combination group and ABI alone was 22.3 and 26.0 months, respectively (HR 1.122, *P* = .2636).^10^ Moreover, the addition of radium‐223 to ABI failed to show an improvement of OS compared to ABI alone, but rather a worse OS (Median OS 30.7 vs 33.3 months; HR 1.195, *P* = .128). These results of ERA 223 trial could be justified with several reasons. Spratt et al stated that the synergistic inhibitory effect on osteoblasts from ABI, combined with the osteoclastogenic effects of androgen deprivation, glucocorticoids, and Ra‐223 increased the bone fracture risk.^22^ Cursano et al provided another possible reason to be the anabolic and antiresorptive effect of ABI on the bone.^23^ As ABI inhibits osteoclastic function and promotes osteoblastic differentiation,^24^ the abnormal deposition of radium‐223 could accumulate in a healthy bone, and lead to cell and tissue damage. Another opinion to explain this result is the excessive dose of prednisone administered in the trial. The glucocorticoids are known to be associated with bone loss and fragility even at low doses.^25^, ^26^ Therefore, Dalla Volta et al suggested that if the dose of prednisone had been lower, such as 5 mg daily as in the hormone‐sensitive setting, and administration of bone resorption inhibitors had been preplanned in the trail, the result might have been different.^27^

The ERA 223 trial also demonstrated that Ra‐223 with ABI increased the frequency of bone fracture compared to ABI alone. The ERA 223 trial reported that among 392 patients of Ra‐223 with ABI group and 394 patients of ABI alone group, the bone fracture occurred in 103 (26.3%) patients including 36 (9.2%) patients with grade 3‐4 fracture and 37 (9.4%) patients including 12 (3.0%) patients with grade 3‐4, respectively.^11^ In the Japanese ERA 223 subgroup, the incidence of fracture was 23% of patients including 3.6% of patients with grade 3 and similar to results in the overall population of the ERA 223 trial.^28^ In our study, the bone fracture was known to have occurred in only one patient (4.2%) with grade 3 in the combination group. The distribution of patients with EOD 1‐2 and 3‐4 was 77% and 23%, respectively, in the combination group of the ERA 223 trial, while, 75% and 25%, respectively, in the combination group of our study. There was no difference of EOD distribution between the ERA 223 trial and our study. In the ERA 223 trial, BMAs such as zoledronic acid and denosumab were used in 39% of patients on Ra‐223 with ABI, while 41.7% of patients received BMAs in the combination group of our study. There was also no difference of BMAs use between the ERA 223 trial and our study. The reasons for high frequency of bone fracture in the combination group of ERA 223 trial compared to our study remains unclear; however, the possibility of underestimation of grade 1‐2 bone fracture in our study cannot be denied. The phase III, EORTC 1333/PEACE III trial (NCT02194842) evaluated whether Ra‐223 with ENZ improved the radiological progression‐free survival compared to ENZ alone among men with bone metastatic CRPC.^29^ Considering the results of the ERA 223 trial, the PEACE III trial was amended to mandate use of BMAs in all patients. Indeed, the BMAs use decreased the frequency of bone fracture among patients treated by Ra‐223 with ENZ from 33% (95% CI 19‐50) to 3% (95% CI, 0‐16). These reports suggest that BMAs might play an important role in preventing fractures among patients treated by Ra‐223 with ARTA.

In our study, both EOD classifications and pain at baseline were significant prognostic factors for the OS. Among the patients treated with Ra‐223 for bone metastatic CRPC, the ALP levels at the baseline has been reported as a prognostic factor from various reports^30^ ^,^ ^14^, ^31^ and recently, the bone metastatic burden measured by the bone scan index (BSI) also has been reported to be of prognostic value.^32^ The BSI is a more objective imaging biomarker rather than the EOD, a subjective measure, and is already used for its prognostic value in a prospective study for patients with mCRPC.^33^ The pain at baseline has also been a significant prognostic factor for the OS among patients treated with Ra‐223.^14^ These factors at baseline are important to ensure a good prognosis for patients treated with Ra‐223.

In our study, the HR of the univariate and multivariate analyses of concurrent nonuse of BMAs was 0.65 and 3.50, respectively. BMAs was used in the 24 out of 43 (55.8%) patients with pain. On the contrary, only seven out of 36 (19.4%) patients without pain was used BMAs. In other words, the patients with aggressive disease received BMAs than the patients with nonaggressive disease. Therefore, concurrent nonuse of BMAs seemed to be a favorable (HR < 1.0) prognostic variable, as determined using univariate analysis. This can be attributed to the contrary results between univariate and multivariate analysis.

There are several limitations that we need to consider as well. First, it was a retrospective study with a small number of patients and a short follow‐up period. The baseline PSA and ALP levels were significantly higher in the single group than in the combination group. To adjust the cofounding factor including the PSA and ALP levels, we performed a multivariate analysis and found that concurrent use of second‐generation ARTA has no influence on OS among men treated with Ra‐223. However, it cannot be denied that the differences in baseline variables between the two groups could influence our results because of the retrospective nature of the study. Second, the use of BMAs was decided by the attending physician and the timing of introduction and dosing interval differed between cases. Finally, our study included only Japanese patients; thus, our data cannot be generalized to the global population.

Our study showed that the concurrent use of second‐generation ARTA with Ra‐223 did not improve the OS compared with the use of Ra‐223 alone among Japanese patients with bone metastatic CRPC, even though the combination group had better tendency of survival. Bone fracture occurred in only one patient (4.2%) in the combination group; and thus, the combination therapy seemed well tolerated in our cohort. The ongoing phase III trial of Ra‐223 plus ENZ vs ENZ alone (NCT0219482) is expected to confirm prolonged survival and safety among patients with mCRPC. Until then, Ra‐223 combined with ARTA is not recommended for patients with bone metastatic CRPC.

## CONFLICTS OF INTEREST

The authors have nothing to disclose.

## Supporting information

Fig S1
