# Supplementary material for: Prognosis and safety of radium‐223 with concurrent abiraterone acetate or enzalutamide use for metastatic castration‐resistant prostate cancer: Real‐world data of Japanese patients
Source: BJUI Compass. 2020 Sep 5;2(1):31–8. doi: 10.1002/bco2.42 (PMC8988769; doi:10.1002/bco2.42)
Supplement: Supplementary file 1 — Fig S1 [file BCO2-2-31-s001.pptx]

## Slide 1
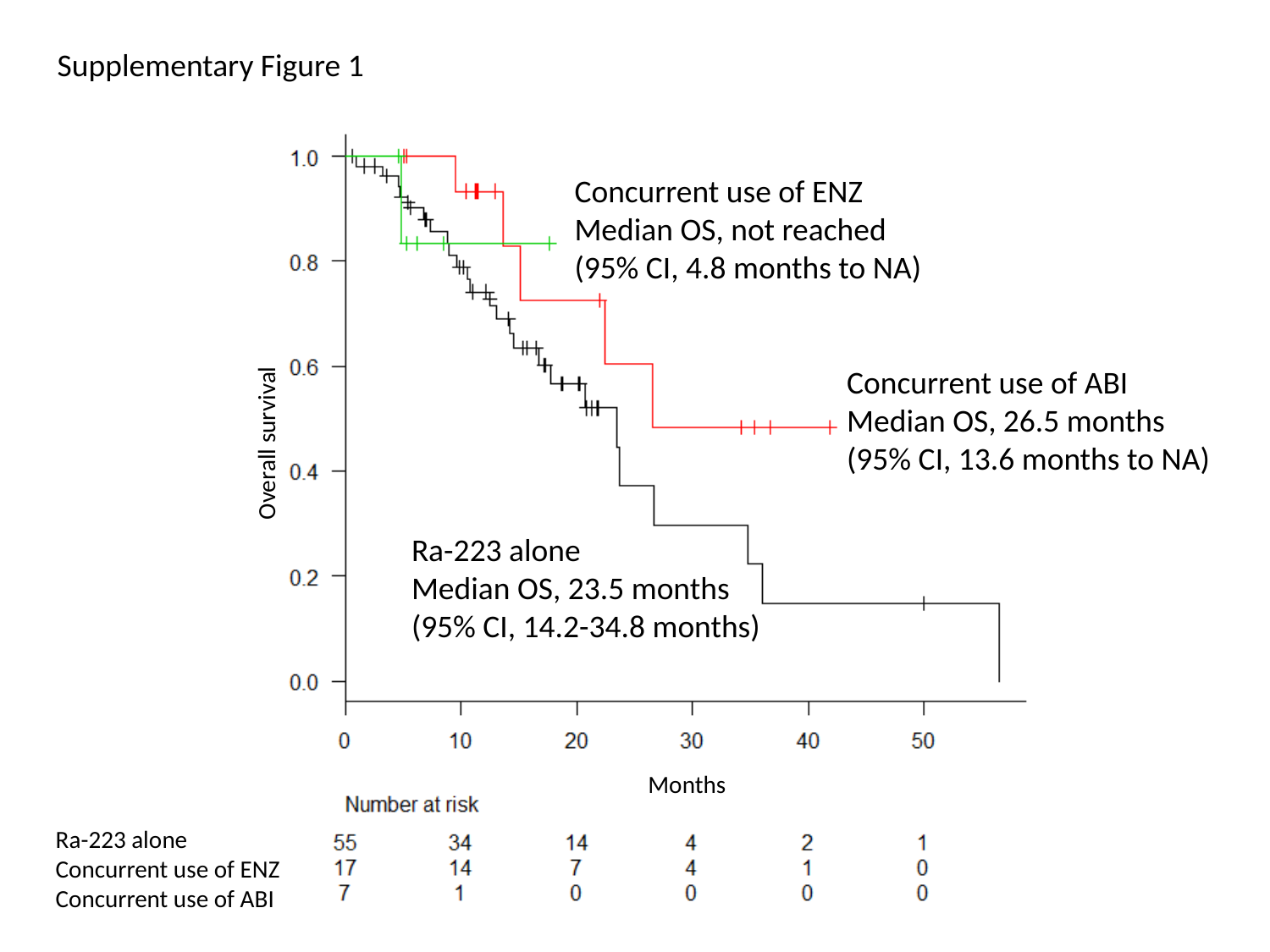

Supplementary Figure 1
Concurrent use of ENZ
Median OS, not reached
(95% CI, 4.8 months to NA)
Concurrent use of ABI
Median OS, 26.5 months
(95% CI, 13.6 months to NA)
Overall survival
Ra-223 alone
Median OS, 23.5 months
(95% CI, 14.2-34.8 months)
Months
Ra-223 alone
Concurrent use of ENZ
Concurrent use of ABI
